# Response of rhizosphere microbial community characteristics and ecosystem multifunctionality to the addition of crude oil in *Achnatherum splendens* and *Pennisetum alopecuroides*

**DOI:** 10.3389/fmicb.2025.1553070

**Published:** 2025-04-15

**Authors:** Ying Wei, Yukun Zhu, Lili Nian, Liqun Yang, Ming Yue, Zhuxin Mao, Lijuan Li

**Affiliations:** ^1^Xi'an Botanical Garden of Shaanxi Province/Institute of Botany of Shaanxi Province, Xi'an, China; ^2^Shaanxi Engineering Research Centre for Conservation and Utilization of Botanical Resources, Xi’an, China; ^3^Shaanxi Provincial Dongzhuang Water Conservancy Engineering Co., Ltd., Xi'an, China; ^4^Institute of Soil, Fertilizer and Water-Saving Agriculture, Gansu Academy of Agricultural Sciences, Lanzhou, China; ^5^Shaanxi Provincial Water Resources Information Education and Promotion Center, Xi'an, China

**Keywords:** *Achnatherum splendens*, *Pennisetum alopecuroides*, ecological network, crude oil addition, ecosystem multifunctionality

## Abstract

This study aimed to reveal the effects of crude oil addition on the characteristics of soil microbial communities and ecosystem multifunctionality in *Achnatherum splendens* and *Pennisetum alopecuroides*. Specifically, it explored how crude oil addition influences the relationship between soil nutrient regulation, microbial community characteristics, and ecosystem multifunctionality. The results indicated that as crude oil addition increased, the Shannon index and Chao1 index for soil bacteria and fungi in both *Achnatherum splendens* and *Pennisetum alopecuroides* increased. Conversely, while the Shannon index for soil archaea in both species increased, the Chao1 index decreased. The ecological network analysis indicated that a strong collaborative relationship existed between species in the soil bacterial communities of *Achnatherum splendens* and *Pennisetum alopecuroides* exposed to 10 g/kg crude oil, as well as between species in the soil fungal and archaeal communities of *Achnatherum splendens* exposed to 40 g/kg crude oil. A strong collaborative relationship was also observed between species in the soil fungal and archaeal communities of *Pennisetum alopecuroides* exposed to 10 g/kg crude oil. The bacterial and fungal communities exerted a significant direct negative regulatory effect on the soil ecosystem multifunctionality of *Achnatherum splendens* and *Pennisetum alopecuroides*, while the archaeal communities had a significant direct positive regulatory effect. Additionally, the multifunctionality index of the soil ecosystem in *Achnatherum splendens* and Pennisetum showed a significant decline with increasing crude oil addition. This is likely due to the higher toxicity of high-concentration crude oil, which negatively impacts the soil biological community, leading to reduced biodiversity and disruptions in nutrient cycles. This study explores the characteristics of bacterial, fungal, and archaeal communities and ecosystem multifunctionality under different levels of crude oil, which can provide theoretical support for evaluating the restoration of *Achnatherum splendens* and *Pennisetum alopecuroides* from crude oil pollution.

## 1 Introduction

Soil is a vital component of the Earth’s ecosystem, and serves as the foundation for agricultural development. It is a crucial environmental resource for human beings and other livings organisms. Soil quality is intricately linked to the future of human society (Loyal et al., 2023). In recent years, with the rapid advancement of global industrialization, oil pollution has become a critical issue requiring immediate attention in the field of environmental science. Oil leaks, spills, and associated industrial activities have led to severe contamination of ecological components such as soil and water (Ukhurebor et al., 2021). According to statistics, global oil spills resulting from oil extraction, transportation, and storage amount to millions of tons annually, significantly impacting ecosystems (Aa et al., 2022). The pollution not only directly poses threats to the survival and reproduction of organisms but also has profound implications for the structure and function of ecological systems (Chakraborty and Chakraborty, 2021). Plants, as significant components of ecosystems, play a crucial role in environmental restoration and ecological balance. They not only offer habitats and food sources for animals but also contributed to soil nutrient cycling and water regulation (Jiang et al., 2022). Recent studies have shown that oil pollution has significant impacts on plant growth, development, and physiochemical processes (Molina and Segura, 2021). Therefore, investigating the effects of oil pollution on plant growth and its rhizosphere microbial community can provide theoretical and practical significance for developing effective ecological restoration strategies.

In environments where soil is contaminated with crude oil, various plants showed differing degrees of adaptability. *Pennisetum alopecuroides* and *Achnatherum splendens* are key species in arid ecosystems in China and are known to grow in contaminated soils and promote ecosystem recovery (Liang et al., 2023; Liu et al., 2016). Both *A. splendens* and *P. alopecuroides* not only exhibit high tolerance to changes in soil moisture, nutrient composition, and salinity, but they also have rapid growth in polluted environments while enhancing the restoration of ecosystem functions (Qi et al., 2021). These plants effectively adapt to crude oil-contaminated soils by absorbing and sequestrating pollutants through their root systems, contributing to the improvement of soil quality (Zhao et al., 2024). Moreover, *A. splendens* and *P alopecuroides* enhance soil permeability through their well-developed root systems, thereby promoting water and nutrient flow while increasing water retention capacity, thus creating a more suitable rhizosphere microenvironment for surrounding organisms (Qu et al., 2023; Sastrawan and Futra, 2020). Additionally, the release of root exudates during their growth stimulates the proliferation of beneficial microorganisms, further enhancing soil ecology (Jin et al., 2023). *A. splendens* and *P alopecuroides* provide habitats for diverse microbial communities within their rhizospheres, which not only promote plant nutrient uptake but also strengthen plant stress resistance capabilities (Molnár et al., 2023). Through interactions with rhizosphere microbes, these plants enhance both soil microbial diversity and functional diversity resulting in a healthy ecosystem (Herrera Paredes and Lebeis, 2016). In cases specifically related to crude oil pollution, this mutual relationship becomes even more critical as it helps plants withstand the negative impacts brought about by contamination.

Soil microorganisms, including bacteria, fungi, archaea, and viruses, play a crucial role in the transformation and utilization of organic matter as well as the degradation of organic waste. Additionally, they are essential for metabolic processes involving inorganic substances (Neemisha and Sharma, 2022). Their active participation in soil system formation and evolution has been extensively studied to highlighting their sensitivity as pollution indicators and their importance as parameters for assessing soil productivity (Aponte et al., 2020). Bacteria and fungi primarily facilitate the degradation of complex organic molecules into simpler compounds that can be utilized by plants and other microorganisms (Yarwood, 2018). Archaea play a pivotal role in environmental sustainability through methane consumption which effectively reduces atmospheric methane levels (Evans et al., 2019). Soil viruses significantly influence the maintenance of ecological balance and the promotion of biodiversity by infecting bacteria, archaea, and fungi within the soil ecosystem. These interactions have profound effects on the structure and function of soil microbial communities (Kuzyakov and Mason-Jones, 2018). Consequently, investigating the dynamic changes in soil microorganisms under crude oil pollution is essential for evaluating soil health and supporting ecological restoration efforts alongside pollution control strategies. Future research should prioritize exploring mechanisms underlying microbial community recovery with potential applications in environmental remediation to promote ecological restoration of contaminated soils.

Soil microorganisms play a crucial role in ecosystem restoration. Studies have shown that microbial community structures in oil-contaminated areas are highly complex, with various interactions among microbial populations, including symbiosis, competition, and predation, collectively influencing pollutant degradation efficiency. Co-occurrence network analysis enables researchers to uncover the ecological relationships within microbial communities and their response mechanisms to environmental changes. By analyzing species correlations and constructing microbial symbiotic networks, key species and their functional roles within the community can be identified. Research has demonstrated that key bacterial groups, such as *Pseudomonas* and *Acinetobacter*, occupy central positions in hydrocarbon degradation networks, forming close symbiotic relationships with other microorganisms and significantly impacting pollutant degradation processes (Centler et al., 2020; Truskewycz et al., 2019). Additionally, co-occurrence network analysis provides critical insights into how environmental factors (e.g., pH, temperature, and nutrient availability) regulate the structure and function of microbial communities (Li et al., 2022). These findings offer theoretical support for optimizing oil pollution remediation strategies and provide a novel perspective for advancing microbial ecology.

Soil microorganisms are an essential component of ecosystems and play a pivotal role in driving various ecosystem functions, including the enhancing plant productivity and regulation of soil nutrient cycling and energy flow (Zhang et al., 2020). Ecosystem multifunctionality (EMF) denotes the capacity of ecosystems to simultaneously provide multiple ecosystem functions and services (Hector and Bagchi, 2007). As a comprehensive metric for evaluating diverse ecological functions, EMF holds significant importance in comprehending the structure and function of ecosystems. In recent years, research on EMF have increasingly focused on in ecology; however, there is remain poorly understood regarding the mechanisms underlying the contribution of soil microorganisms to EMF within oil-polluted grasslands undergoing ecological restoration. Indoor controlled experiments conducted in natural ecosystems have shown a positive correlation between soil microbial diversity and EMF (Wagg et al., 2014). Therefore, as degraded ecosystems experience a recovery in soil microbial diversity, their corresponding ecosystem functions also experience a recovery. In addition, researchers have discovered that microbial communities in natural environments do not operate in isolation; instead, they form intricate ecological networks through processes such as material cycling, energy flow, and information transmission. These network characteristics are essential in maintaining ecosystem functionality (Fuhrman, 2009; Zhou et al., 2010). Although co-occurrence network analysis has certain limitations in precisely depicting the true ecological connections between species (Freilich et al., 2018), it still provides a valuable method to understanding the structure of microbial communities and interactions among species. This approach aids in characterizing complex relationships that cannot be directly observed (Banerjee et al., 2018). Previous studies on soil amended with crude oil have predominantly concentrated on physicochemical and biological indicators of soil quality, microbial community diversity and composition (Chen et al., 2023; Zhuang et al., 2023), however, there is a notable lack of comprehensive research on co-occurrence network patterns or structural characteristics and soil multifunctionality under different levels of crude oil contamination.

The study was conducted at the Xi’an Botanical Garden in Xi’an, Shaanxi Province, China. It utilized *A. splendens* seeds collected from Fengyukou, Chang’an District, Shaanxi Province and *P. alopecuroides* seeds collected from Ejin Horo Banner, Ordos City, Inner Mongolia. By simulating crude oil pollution, it investigated the impact of varying concentrations of crude oil pollution on the growth of *A. splendens* and *P. alopecuroides* while analyzing changes in rhizosphere microbial community structure. While different levels of crude oil pollution (0 g/kg, 10 g/kg, 40 g/kg) and utilizing methods such as biomass determination, analysis of rhizosphere soil enzyme activity, and metagenomic analysis, it systematically evaluated the effects of crude oil pollution on *A. splendens* and *P. alopecuroides* as well as their rhizosphere microorganisms. Furthermore, this study also investigated the interaction mechanisms between *A. splendens* and *P. alopecuroides* with rhizosphere microorganisms, specifically focusing on how these microorganisms modulate plant physiological and biochemical responses in the presence of crude oil contamination, and delved into the intricate co-occurrence networks within soil microbial communities (including archaea, bacteria, and fungi) and their impact on ecosystem multifunctionality. It is anticipated that this comprehensive investigation will provide a robust theoretical foundation and practical technical support for the ecological restoration of crude oil-contaminated soils, as well as offer scientific guidance for the utilization of *A. splendens* and *P. alopecuroides* in such restoration efforts.

## 2 Materials and methods

### 2.1 Experimental materials

The experiment was conducted in April 2023 at the Xi’an Botanical Garden, located in Shaanxi Province, China. The seeds of *Achnatherum splendens* (Trin.) Nevski were collected from the grasslands in Ejin Horo Banner, Ordos City, Inner Mongolia in August 2020. The seeds of *Pennisetum alopecuroides (L.) Spreng*. were gathered from Fengyukou, Chang’an District, Shaanxi Province in October 2022. The crude oil utilized was sourced from Ansai Oilfield in Yan’an City, Shaanxi Province. Soil samples for the experiment were collected from the designated planting area within the Xi’an Botanical Garden. The topsoil layer (0–20 cm) was dried and sieved to remove impurities, then mixed with river sand at a volume ratio of 3:1. After thorough mixing, the crude oil was added to the mixture dissolved in petroleum ether at a volume ratio of 1:1 and uniformly incorporated into the soil sample. To ensure homogenization of pollutants and a significant impact on soil biochemical properties, which serve as indicators of soil stress on *A. splendens* and *P. alopecuroides*, the contaminated soil samples underwent natural homogenization treatment for 30 days under shaded conditions to simulate recently polluted surface soils.

### 2.2 Experimental design

The current study aimed to simulate the soil oil pollution scenario in the oil extraction area of Yan’an City, Shaanxi Province, China integrating insights from prior studies (Xia et al., 2022; Zhao et al., 2024). The crude oil pollution levels were established at 0 g/kg (CK), 10 g/kg (F10), and 40 g/kg (F40) with three replicates for each treatment level. The 0 g/kg concentration served as the control group, providing baseline data under natural conditions. The 10 g/kg concentration represented a moderate pollution level commonly observed in real-world environments, allowing for an assessment of typical ecological effects. The 40 g/kg concentration simulated severe pollution conditions and was used to explore soil tolerance thresholds and recovery potential under extreme contamination. For the experiment, seedlings were first grown in seedling trays until they reached the four-leaf stage. Uniformly developed seedlings were then selected and transplanted into 10 cm × 10 cm plastic pots. Once reaching a height of approximately 10 cm, the seedlings underwent rinsing with running water before being transplanted into plastic pots measuring 20 cm × 15 cm for crude oil stress treatment. Each pot contained 1.6 kg of soil exposed to crude oil, with three replicates for each treatment level. The experimental setup was placed within a greenhouse and maintained under identical moisture management conditions without fertilization or weed removal throughout the five-month cultivation period from June 12th to November 2nd. The core of this experimental design is to simulate soil pollution under real-world conditions by applying crude oil contamination at varying concentrations. This approach aims to investigate the effects of crude oil pollution on the growth of *A. splendens* and *P. alopecuroides* as well as their tolerance mechanisms. Throughout the experiment, all treatments adhered strictly to scientific protocols to ensure data accuracy and repeatability, providing a reliable foundation for further research on the ecological impact of soil pollution on plants.

### 2.3 Soil sample collection

After the experiment, plant samples were manually separated into above-ground (stems and leaves) and underground (roots) components. The roots were rinsed with running water, placed in labeled sample bags, and temporarily stored at low temperatures. Soil samples were divided into three portions: one was placed in a sterilized centrifuge tube, packed in a foam box with ice packs, transported to the laboratory, and stored in a −80°C ultra-low temperature freezer for total soil DNA extraction. The second portion was transported to the laboratory and stored in a 4°C refrigerator for soil enzyme activity analysis. The third portion was air-dried at room temperature, sieved to remove impurities, and used to determine soil physicochemical properties, including pH, available phosphorus, available nitrogen, and available potassium.

### 2.4 Determination of soil enzyme activity

The determination of soil enzyme activity was performed using the microplate fluorescence method as described by Frankenberger and Johanson (1983). The fluorescent substance, 4-methylumbelliferone (MUB), was used as the standard control for β-glucosidase (BG), N-acetylglucosaminidase (NAG), cellulose hydrolyzing enzymes (CBH), and acid phosphatase (ACP) activities. Frozen soil samples were thawed at 4°C for 5 days. Then, 1 g of fresh soil with a similar pH to the sample using sodium acetate buffer, which was adjusted with HCl or NaOH. The resulting soil slurry was thoroughly mixed on a magnetic stirrer for approximately 1 min. An aliquot of 200 μL of the soil slurry was transferred to each well of a 96-well microplate using an 8-channel pipette. Each well received 50 μL of the corresponding enzyme reaction substrate. Control groups included sodium acetate buffer, standard control substances, and specific substrates, respectively. The microplate containing the samples was incubated in the dark at 20°C for 4 h. After incubation, all reactions were terminated by adding 10 μL 1.0 M NaOH solution. Readings were immediately taken using an enzyme reader (Synergy H1) with excitation wavelengths set at 365 nm and emission wavelength at 450 nm.

The oxidase activity assay was performed following the method described by DeForest (2009). A plastic bottle containing 1 g of dry weight fresh soil sample was supplemented with 100 mL of sodium acetate buffer solution (pH adjusted to match the soil sample using HCl or NaOH). The mixture was homogenized on a magnetic stirrer for approximately 1 min to obtain a soil slurry. In the negative control wells, 200 μL of buffer solution and 50 μL of substrate (a combination of levodopa at a concentration of 25 mmol·L^−1^ and ethylenediaminetetraacetic acid disodium salt at a concentration of 50 mmol·L^−1^) were added. In the blank wells, 200 μL of soil suspension and 50 μL of buffer solution were added. In the sample wells, 200 μL of soil suspension and 50 μL of substrate were added. For peroxidase analysis, all wells received an additional 10 μL of 0.3% H_2_O_2_ solution at a concentration of 0.3% (10 μL). The microplate was incubated in the dark at 20°C for 20 h, followed by 100 μL of the reaction mixture was transferred to a transparent 96-well plate, and 10 μL of 1 M NaOH solution was added to terminate the reaction (1 mol/L) to terminate the reaction. The absorbance at wavelength 460 nm was measured using a multifunctional enzyme analyzer.

### 2.5 Soil microbial metagenomic sequencing

The total genomic DNA was extracted from the samples using the E.Z.N.A.® Soil DNA Kit (Omega Bio-tek, Norcross, GA, United States). The concentration and purity of the extracted DNA were measured using TBS-380 and a NanoDrop2000 spectrophotometers, respectively. The quality of the DNA extraction solution was evaluated by 1% agarose gel electrophoresis. Subsequently, the extracted DNA fragments were sheared to an average size of approximately 400 bp utilizing Covaris M220 system (Gene Company Limited, China) and then constructed into paired-end libraries. Paired-end libraries were prepared employing the NEXTflexTM Rapid DNA-Seq kit (Bioo Scientific, Austin, TX, United States), where adapters containing complete sequencing primer hybridization sites were ligated to blunt ends of the fragments. Paired-end sequencing was conducted on Illumina NovaSeq/Hiseq Xten platforms (Illumina Inc., San Diego, CA, United States) with NovaSeq Reagent kit /Hiseq X Reagent kit at Aijibaike Biotechnology Co., Ltd. in Wuhan, China.

For sequence quality control and genome assembly, the Majorbio Cloud platform^1^ was utilized alongside the fastp tool (Chen et al., 2018),^2^ which is freely accessible online. This tool efficiently removed adapter sequences, trimmed reads, and filtered out low-quality reads according to specific criteria, including the presence of N bases, a minimum length threshold of 50 bp, and a minimum quality score of Q20 As a result, clean reads were generated. Subsequently, MEGAHIT (Li et al., 2015) (parameters: kmer min = 47, kmer max = 97, step = 10)^3^ was employed to assemble these high-quality reads into contigs using a concise de Bruijn graph approach. The final assembly results comprised contigs with lengths greater than or equal to 300 bp.

Species and functional annotation: Representative sequences from the non-redundant gene catalog were annotated using blastp implemented with DIAMOND v0.9.19, based on the NCBI NR database. Classification annotation was performed using DIAMOND (Buchfink et al., 2015),^4^ employing an e-value cutoff of 1e^−5^. KEGG annotation was conducted using Diamond (Buchfink et al., 2015)^5^ against the Kyoto Encyclopedia of Genes and Genomes database,^6^ applying an e-value cutoff of 1e^−5^.

### 2.6 Calculation of ecosystem multifunctionality

The assessment of ecosystem functions entailed the selection of 12 indicators: soil pH, available potassium, alkaline nitrogen, available phosphorus, polyphenol oxidase activity, hydrogen peroxide enzyme activity, acid phosphatase activity, N-acetylglucosaminidase activity, cellulose hydrolysis enzyme activity, β-glucosidase activity, aboveground biomass and belowground biomass. These indicators effectively encapsulate the regulatory and provisioning functions of ecosystems, including soil nutrient status, organic matter decomposition, and plant productivity. The Multifunctionality Index (Ma et al., 2020; Maestre et al., 2012) was calculated using the mean value method. First, the 12 ecological function indicators are standardized using the following formula: $f_{ij}$ = $(x_{ij}$ - $\min_{ij}$ ) / $(\max_{ij}$ - $\min_{ij}$ ), $f_{ij}$ represents the standardized value of the j th ecosystem function variable for plot i , $x_{ij}$ is the actual measured value of the j th ecosystem function variable for plot i , $\min_{ij}$ is the minimum value of the j th ecosystem function variable across all plots for the same factor, and $\max_{ij}$ is the maximum value of the j th ecosystem function variable across all plots for the same factor.

Ecological Multifunctionality Index (EMF) is calculated using the average value method.


$$EMF_{i}=1/N\sum_{1}^{N}f_{ij}$$

The multifunctionality index of a plot is calculated based on the standardized average value of all variable indicators within the plot; N represents the number of ecosystem functions encompassed by the plot.

### 2.7 Statistical analysis

The data on soil physicochemical properties, bacterial, fungal, and archaeal community compositions were processed using SPSS 26.0 and Excel 2010. One-way analysis of variance (ANOVA) followed by multiple comparisons (LSD method, *p* = 0.05) was conducted to assess significant differences. The Aikebaiker-Sanger cloud platform was utilized for the analysis of bacterial, fungal, and archaeal community compositions and diversities. Redundancy analysis (RDA) was employed to analyze the relationship between bacterial, fungal, and archaeal communities with soil environmental factors. The molecular ecological network was constructed using the Spearman correlation coefficient (≥ 0.5) with a significance threshold of *p* < 0.05. The correlation network was generated using the “igraph” and “psych” packages in R and visualized with Gephi (Version 0.9.2). A partial least squares path model (PLS-PM) was developed using the “plspm” package in R to analyze the causal relationships among bacteria, fungi, archaea, and soil ecosystem multifunctionality. The final model was optimized by iteratively removing non-significant paths to achieve the best fit to the data. Graphs were plotted using CANOCO 5.0 software and further edited in Adobe Illustrator CS6.

## 3 Results

### 3.1 Physicochemical properties

**Figure 1:**
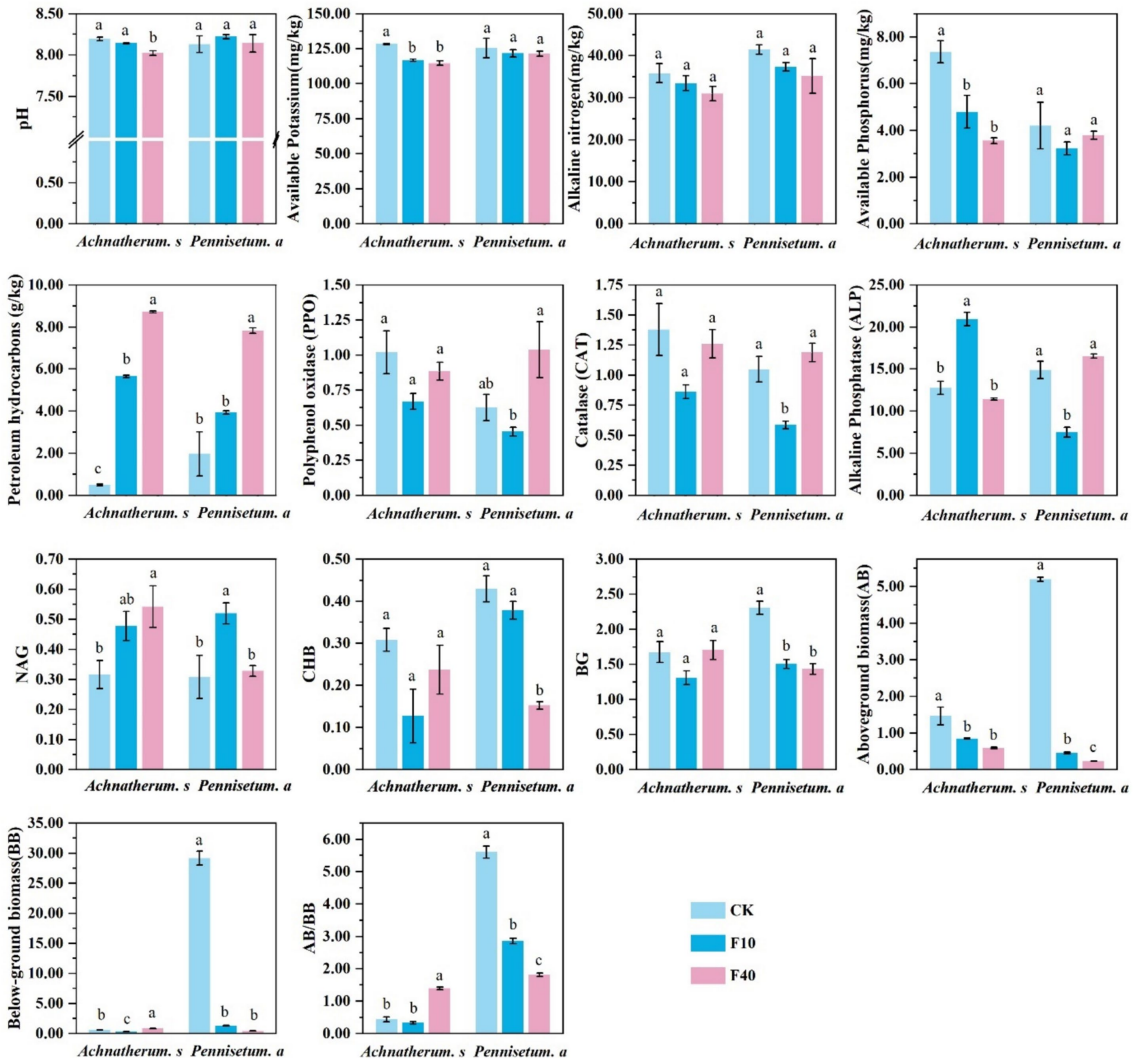
Physical and chemical properties of soil. Different lowercase letters indicate significant differences with a value of *p* < 0.05 based on the ANOVA.CK-0 g/kg, F10-10 g/kg, F40–40 g/kg.

The physicochemical properties of the soil, as depicted in Figure 1, demonstrated significant decreases in pH, available potassium, and available phosphorus after the addition of crude oil to *A. splendens* soil. In contrast, no significant alterations in these parameters were observed in *P. alopecuroides* soil (*p* < 0.05). The addition of crude oil resulted in a significant increase in petroleum hydrocarbon content in both *A. splendens* and *P. alopecuroides* soils (*p* < 0.05). Soil enzyme activity analysis revealed that crude oil addition did not significantly affect polyphenol oxidase (PPO), catalase (CAT), and β-glucosidase (BG) activities in *A. splendens* soil; however, it significantly altered PPO, CAT, and BG activities in *P. alopecuroides* soil (*p* < 0.05). Notably, the activities of PPO, CAT, and BG were significantly lower in the F10 treatment compared to other treatments. In *A. splendens* oil, ALP activity was significantly higher in the F10 treatment than in other treatments (*p* < 0.05); whereas in *P. alopecuroides* soil, ALP activity was significantly lower in the F10 treatment than other treatments (*p* < 0.05). The content of N-acetylglucosaminidase (NAG) was significantly higher in the F40 treatment compared to other treatments in *A. splendens* soil, whereas it was significantly higher in the F10 treatment compared to other treatments in *P. alopecuroides* soil (*p* < 0.05). Analysis of the above-ground to below-ground biomass ratio revealed that with increasing crude oil addition, the ratio significantly increased in *A. splendens* soil but significantly decreased in *P. alopecuroides* soil (*p* < 0.05).

### 3.2 Soil microbial community structure and composition

**Figure 2:**
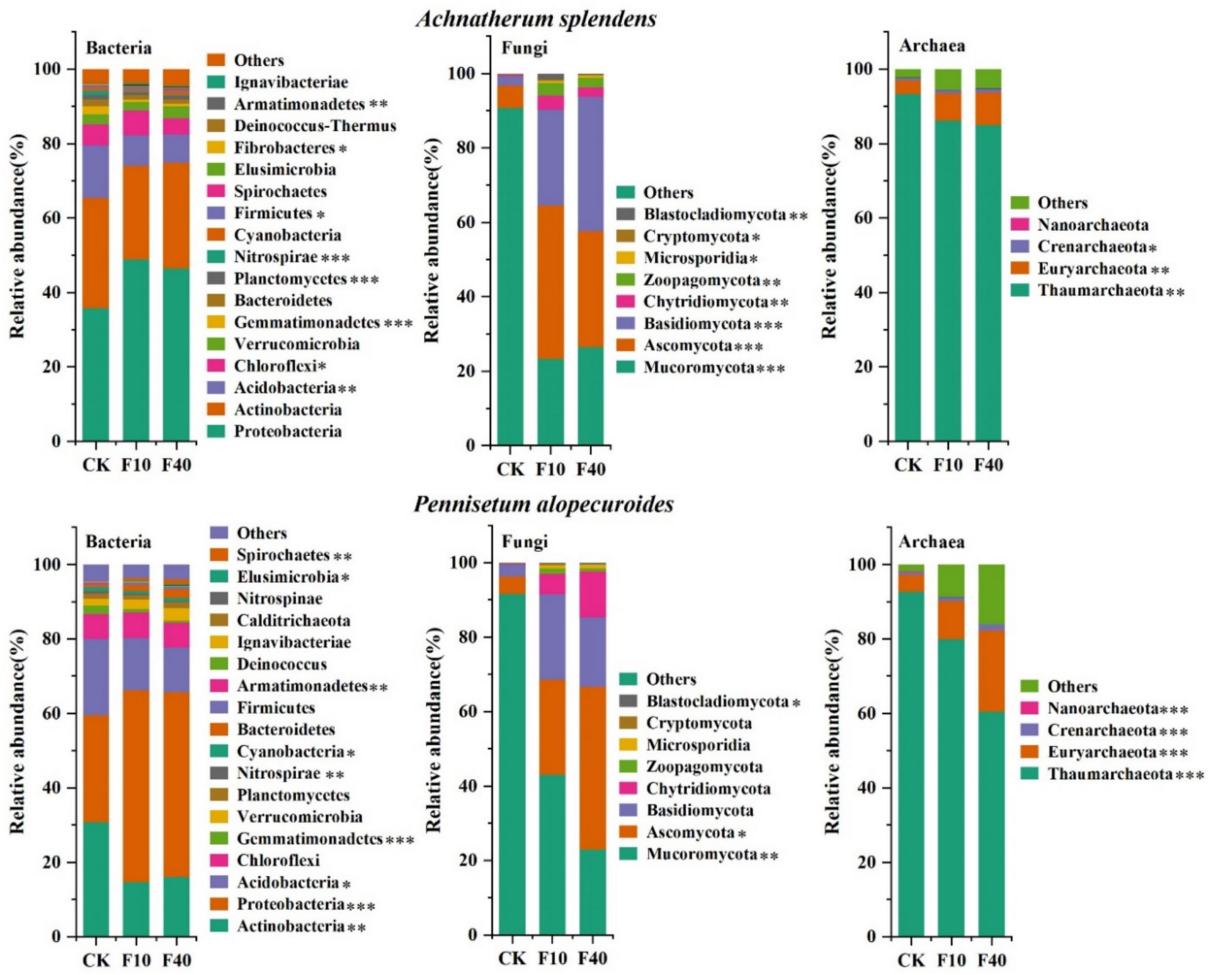
Soil microbial community composition. The asterisk indicated a significant difference between treatments (*p < 0.05, **0.01 < *p* < 0.05, ***p < 0.01).

Through metagenomic sequencing of microorganisms (Figure 2), it was founded that *Proteobacteria*, *Actinobacteria*, and *Acidobacteria* are the predominant phyla in the bacterial community of *A. splendens* and *P. alopecuroides*. The relative abundance of Proteobacteria in *A. splendens* was 79.49% (CK), 82.14% (F10), and 82.37% (F40) across different crude oil concentration, while in *P. alopecuroides*, it was 80.01% (CK), 80.23% (F10), and 77.67% (F40). These findings suggest an increasing trend in the dominance of Proteobacteria in *A. splendens* with higher crude oil additions, whereas for *P. alopecuroides*, a decreasing trend is observed. The analysis of fungal phyla in *A. splendens* and *P. alopecuroides* revealed that *Ascomycota*, *Basidiomycota*, and *Mucoromycota* were the predominant phyla in both grass species. In *A. splendens*, the relative abundance of *Ascomycota* was 99.29% (CK), 90.31% (F10), and 93.71% (F40) across different levels of crude oil addition, while in *P. alopecuroides*, the relative abundance of *Basidiomycota* was 99.47% (CK), 91.44% (F10), and 85.36% (F40). These results indicated a declining trend in the relative abundance of the dominant fungal phyla of *A. splendens* and *P. alopecuroides* with crude oil addition. The analysis of *Thaumarchaeota* in *A. splendens* and *P. alopecuroides* revealed that phylum predominant in both grass species. In *A. splendens*, the relative abundance of *Thamarchaeota* was 93.29% (CK), 86.15% (F10), and 84.95% (F40) across different levels of crude oil addition. For *P. alopecuroides*, the corresponding values were 92.68% (CK), 79 0.94% (F10), and 60 0.50% (F40). These results suggest a decreasing trend in the dominance of *Thaumarchaeota* associated with both grass species as the concentration of crude oil in the soil increases.

**Figure 3:**
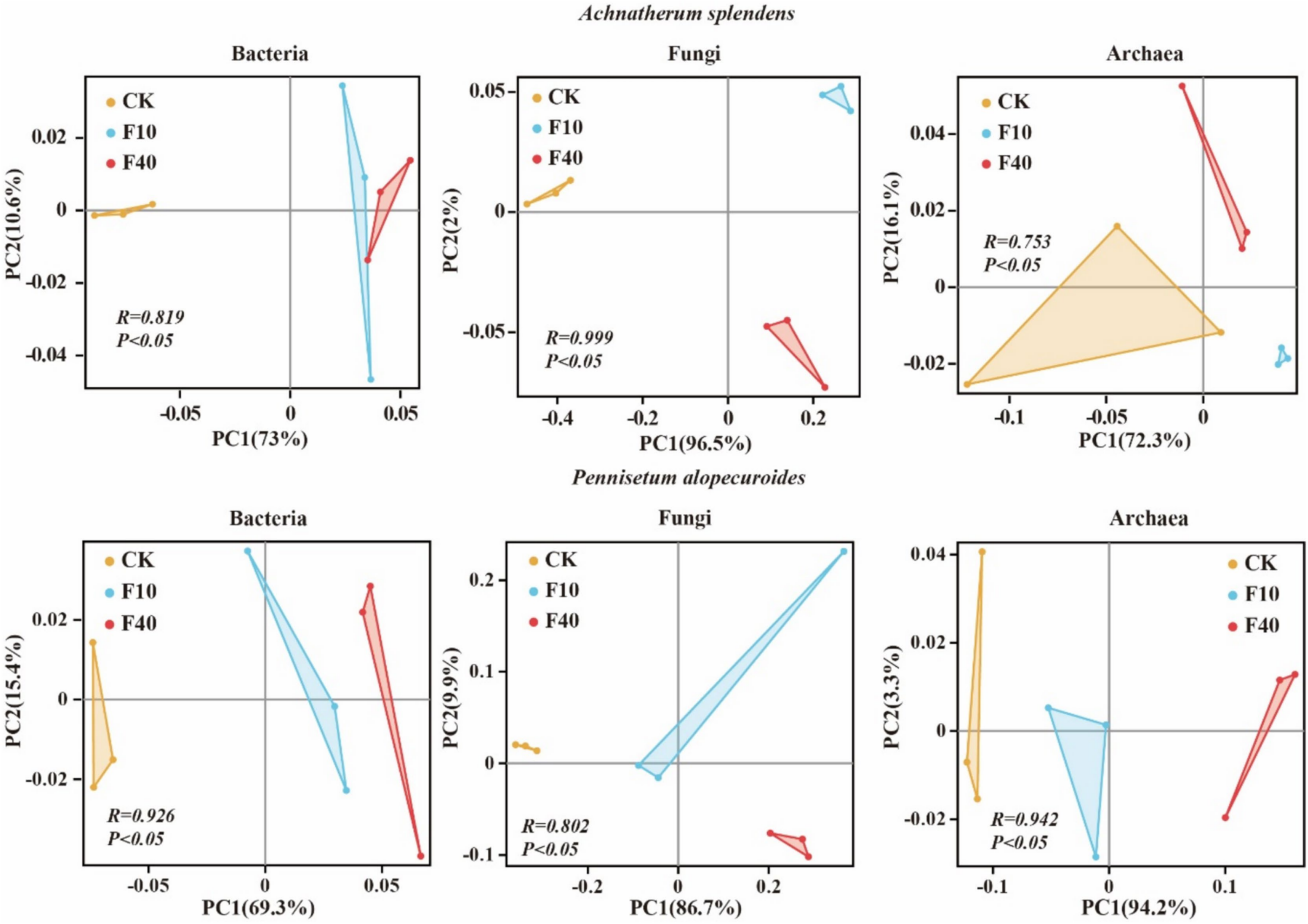
PCA analysis of soil microbial communities. CK-0 g/kg, F10-10 g/kg, F40–40 g/kg.

**Figure 4:**
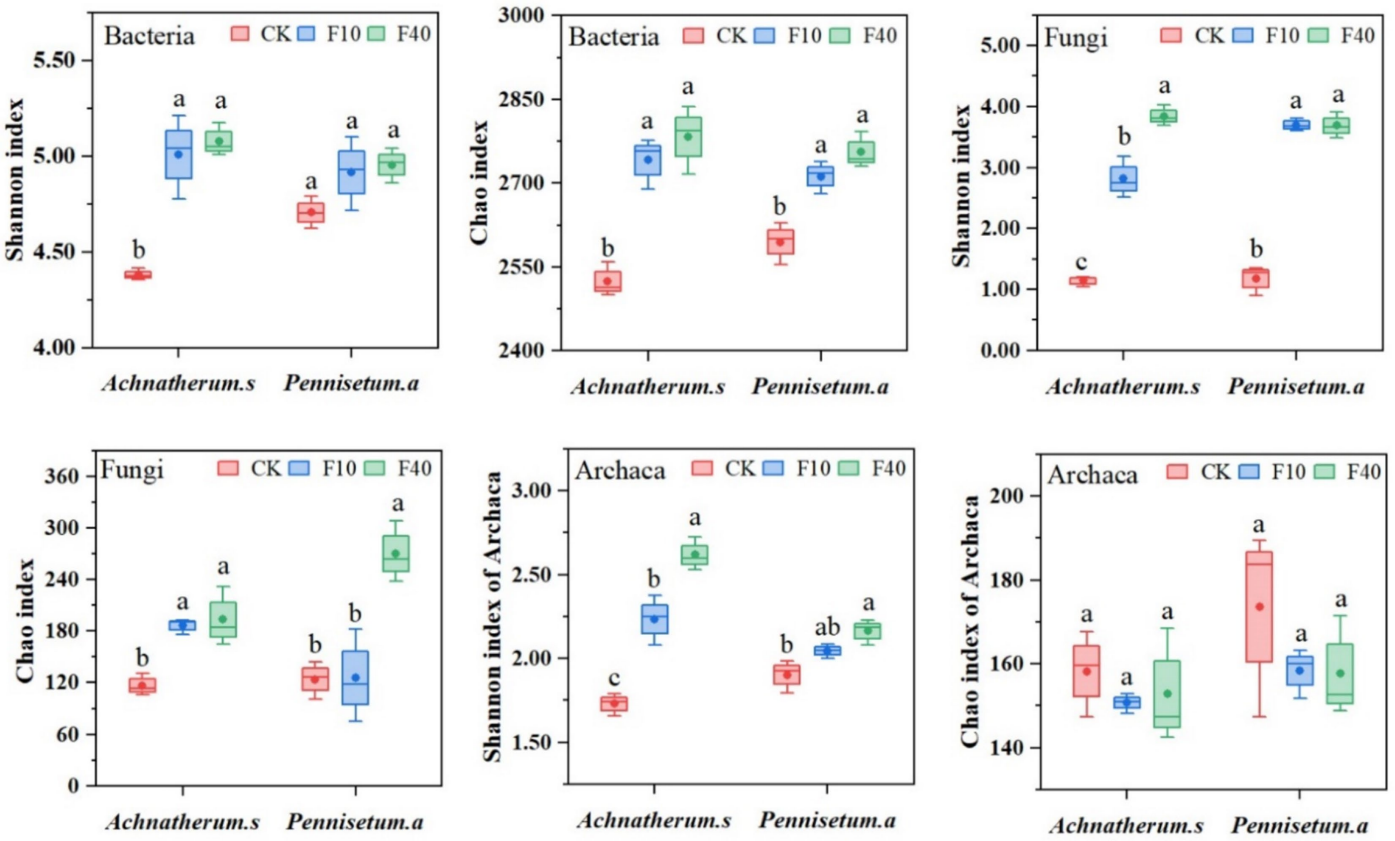
Soil microbial community diversity index. Different lowercase letters indicate significant differences with a value of *p* < 0.05 based on the ANOVA. CK-0 g/kg, F10-10 g/kg, F40–40 g/kg.

The results indicated that PCA analysis that crude oil addition significantly affects the community composition of bacteria, fungi, and archaea (Figure 3). The soil microbial diversity is depicted in Figure 4. Significant differences (*p* < 0.05) were observed in the Shannon and Chao1 index of bacteria, fungi, and archaea between the treatments of *A. splendens* and *P. alopecuroides*. Additionally, as the crude oil concentration increased, a positive trend was noted in the Shannon and Chao index of bacteria and fungi in both *A. splendens* and *P. alopecuroides* soils, with the F40 treatment showed significantly higher diversity indices compared to other treatments (*p* < 0.05). Similarly, the Shannon index of archaea showed an increasing trend in both *A. splendens* and *P. alopecuroides* soils, with the F40 treatment displaying significantly higher diversity index values compared to other treatments (*p* < 0.05), Conversely, the Chao index of archaea demonstrated a decreasing trend, with significantly lower values observed in the F40 treatment compared to other treatments (*p* < 0.05).

### 3.3 KEGG functional annotation and analysis

**Figure 5:**
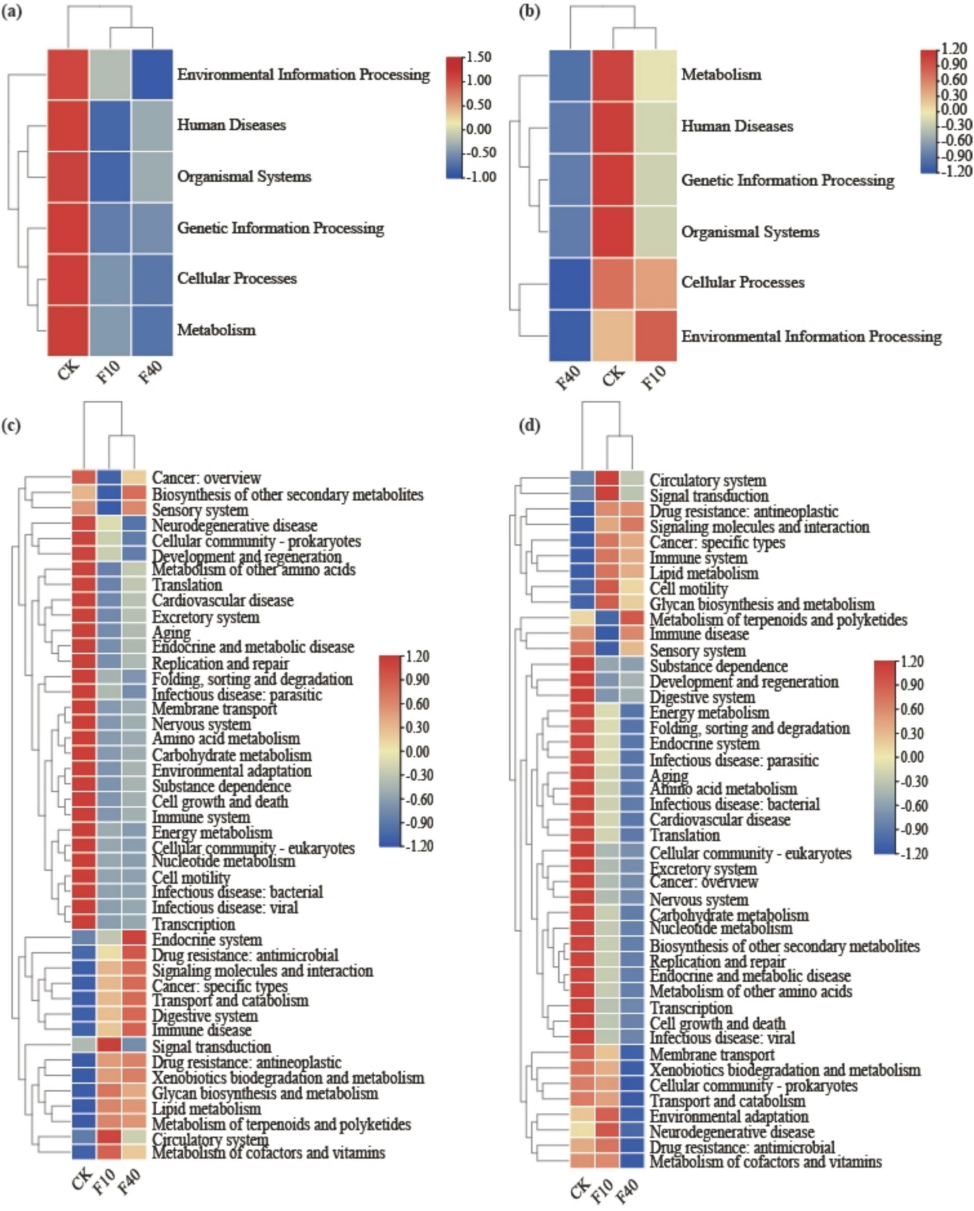
Distinct KEGG functional annotations for community analysis are observed in different treatments, as demonstrated by a heatmap of KEGG level 1 pathway functions in *Achnatherum splendens* **(a)** and *Pennisetum alopecuroides*, **(b)** and a heatmap of KEGG level 2 pathway functions in *Achnatherum splendens* **(c)** and *Pennisetum alopecuroides* **(d)**.

Soil microbial functional genes were annotated using the KEGG database. The gene abundance data pertaining to the primary pathways in soil KEGG for *A. splendens* and *P. alopecuroides* are presented across in six subsystems (Figures 5a,b): Metabolism, Environmental Information Processing, Genetic Information Processing, Cellular Processes, Human Diseases, and Cellular Processes. An increase in crude oil concentration, resulted in a declining trend for all aforementioned subsystems in both *A. splendens* and *P. alopecuroides*. Secondary functional gene annotations via KEGG further indicated that Carbohydrate Metabolism, Amino Acid Metabolism, Energy Metabolism, and Aging showed a decreasing trend with increasing crude oil levels, whereas Metabolism of Terpenoids and Polyketides, Drug Resistance Antineoplastic, Signaling Molecules and Interaction, and Immune Disease showed an increasing trend (Figures 5c,d).

### 3.4 Soil microbial community synteny model

**Figure 6:**
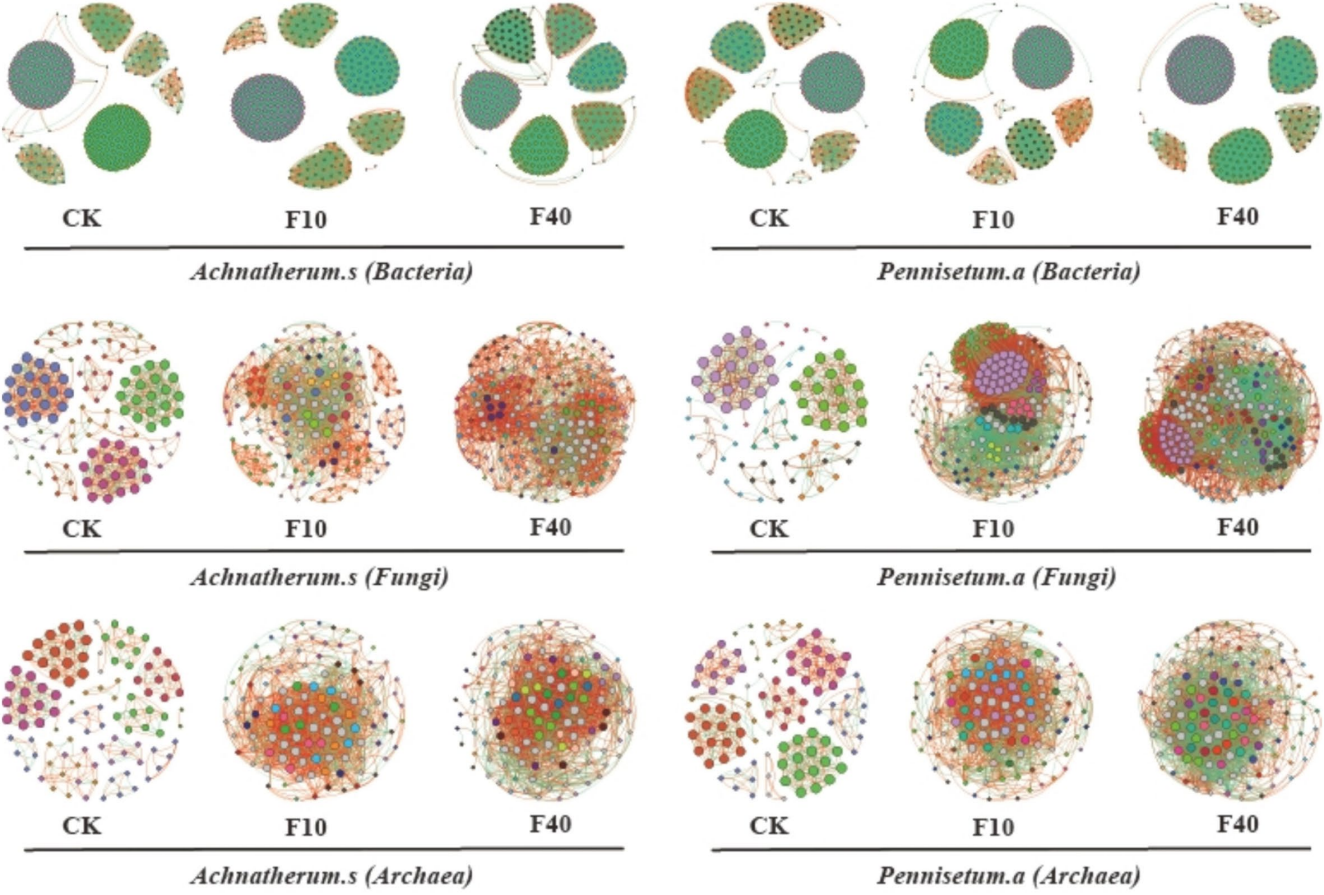
Analysis of collinear network of soil microbial communities. Node size represents degree (or the number of nodes connected to it). The red connection cable is a positive connection, and the green connection cable is a negative connection. The number of nodes is colored according to different categories. Different colored nodes represent different module.

Different levels of crude oil addition significantly influenced the characteristics of co-occurrence networks among soil microorganisms (Figure 6; Tables 1, 2). Network analysis of bacterial communities in *A. splendens* and *P. alopecuroides* soils demonstrated that crude oil addition substantially increased the number of edges and nodes in their co-occurrence networks compared to the control group, indicating enhanced microbial interactions and network complexity. Furthermore, the proportion of positive correlation edges was higher, while negative correlation edges were lower, in the F10 treatment compared to other treatments for both *A. splendens* and *P. alopecuroides*, suggesting a strong cooperative relationship among bacterial species. Network analysis of fungal and archaeal communities in *A. splendens* and *P. alopecuroides* soils revealed that crude oil addition significantly increased the number of edges and nodes in their co-occurrence networks compared to the control group, signifying enhanced complexity and more intricate interactions within the soil fungal and archaeal co-occurrence networks. Additionally, the F40 treatment exhibits a higher proportion of positive correlations and a lower proportion of negative correlations compared to other treatments, indicating a robust cooperative relationship between fungal and archaeal species within the community.

**Table 1:** Topological parameters of the soil microbial co-occurrence network of *Achnatherum splendens*.

Topological parameters	Bacteria			Fungi			Archaea		
	CK	F10	F40	CK	F10	F40	CK	F10	F40
Nodes	297	296	299	115	151	194	206	115	125
Links	10,855	10,684	6,883	630	1,485	2,437	354	1,474	1,727
Positive links (%)	51.84	56.51	50.73	66.51	71.99	78.74	59.32	70.15	78.00
Negative links (%)	48.16	43.49	49.27	33.49	28.01	21.25	40.68	29.85	22.00
Average degree	73.098	72.189	46.04	10.957	19.669	25.124	6.874	25.635	27.632
Modularity	0.598	0.524	0.801	0.773	0.416	0.441	0.855	0.213	0.206
Graph density	0.247	0.245	0.154	0.096	0.131	0.13	0.067	0.225	0.223

**Table 2:** Topological parameters of soil microbial co-occurrence network of *Pennisetum alopecuroides.*

Topological parameters	Bacteria			Fungi			Archaea		
	CK	F10	F40	CK	F10	F40	CK	F10	F40
Nodes	300	300	298	80	194	230	103	118	128
Links	7,980	8,603	12,239	366	4,429	4,562	447	1,735	2,043
Positive links (%)	52.82	54.52	51.64	50.00	85.35	81.26	67.34	70.78	69.65
Negative links (%)	47.18	45.48	48.36	50.00	14.65	18.74	32.66	29.22	30.35
Average degree	53.2	57.35	82.14	9.15	45.66	39.67	8.68	29.41	31.92
Modularity	0.699	0.704	0.451	0.68	0.286	0.419	0.835	0.179	0.146
Graph density	0.178	0.194	0.277	0.116	0.237	0.173	0.085	0.251	0.251

### 3.5 Multifunctionality of soil ecosystems

**Figure 7:**
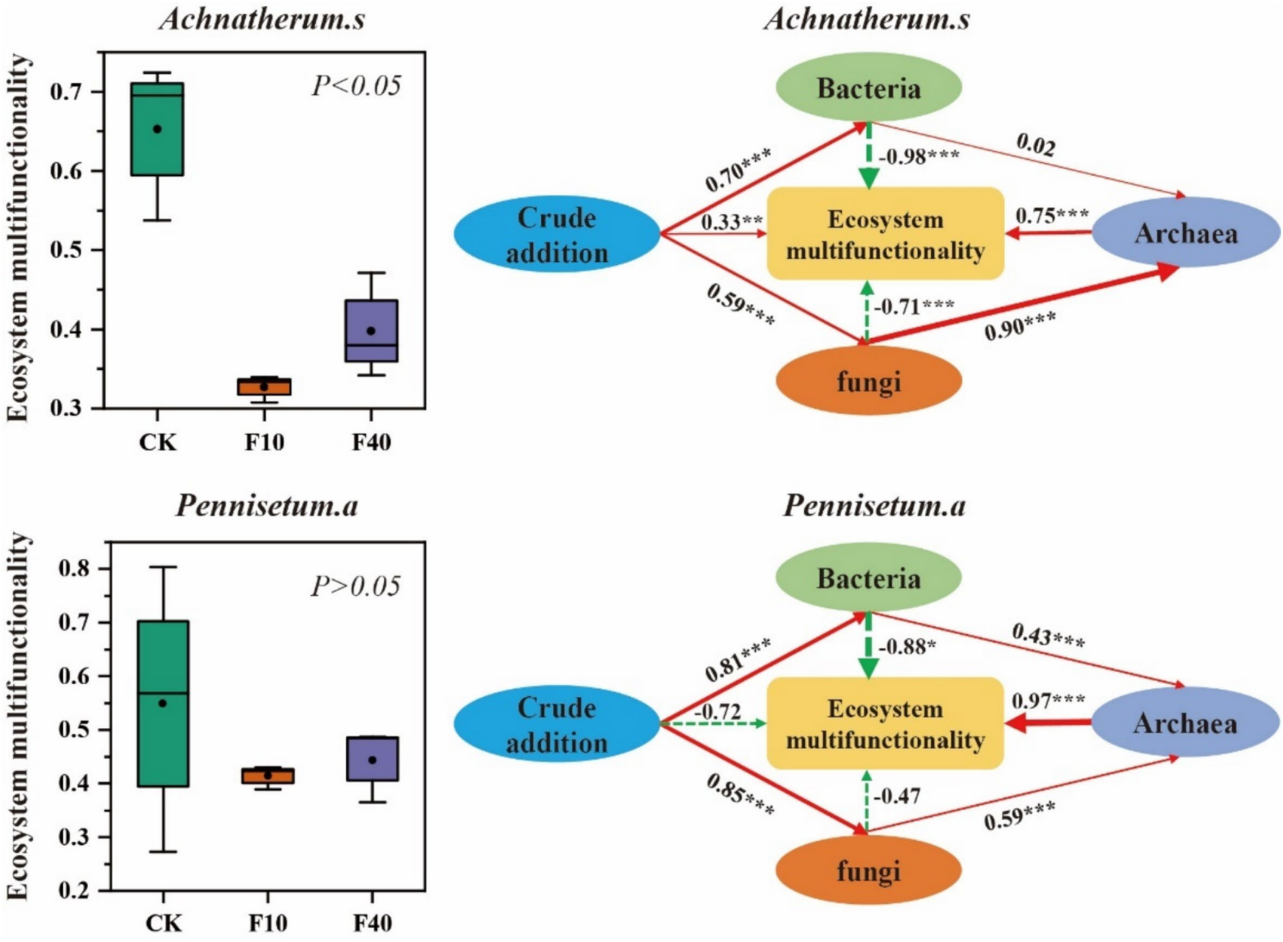
Soil ecosystem multifunctionality. In structural equation model, the red solid arrows represent significant positive correlation paths, the green dashed arrows represent negative correlation paths, and the numbers on the arrows represent standardized path coefficients, in addition, (*Achnatherum splendens*: *X*^2^ = 36.70, GFI = 0.88, RMSEA = 0.06. *Pennisetum alopecuroides*: *X*^2^ = 28.64, GFI = 0.96, RMSEA = 0.03).

Based on the findings from Figure 7, it is evident that the soil ecosystem multifunctionality index of *A. splendens* and *P. alopecuroides* declines with increasing crude oil addition. Notably, the F10 treatment shows a significantly lower soil ecosystem multifunctionality index compared to other treatments (*p* < 0.05). Furthermore, a path model was constructed to comprehensively examine the influence of bacterial, fungal, and archaeal communities on soil ecosystem multifunctionality. The addition of crude oil has a direct positive impact on the soil ecosystem multifunctionality of *A. splendens* while it exerts a direct negative effect on *P. alopecuroides*. Additionally, crude oil addition significantly alters the composition of bacterial and fungal communities, which in turn affects the composition of archaeal community. There is a direct positive correlation between bacterial and fungal communities in relation to archaeal community dynamics. Bacterial, fungal, and archaeal communities have significant direct effects on the soil ecosystem multifunctionality of *A. splendens* and *P. alopecuroides*. Specifically, bacterial and fungal communities exhibit significant direct negative regulation on the soil ecosystem multifunctionality of both grass species. Whereas the while archaeal community demonstrates significant direct positive regulation.

### 3.6 Relationship between soil microbial communities and environmental factors

**Figure 8:**
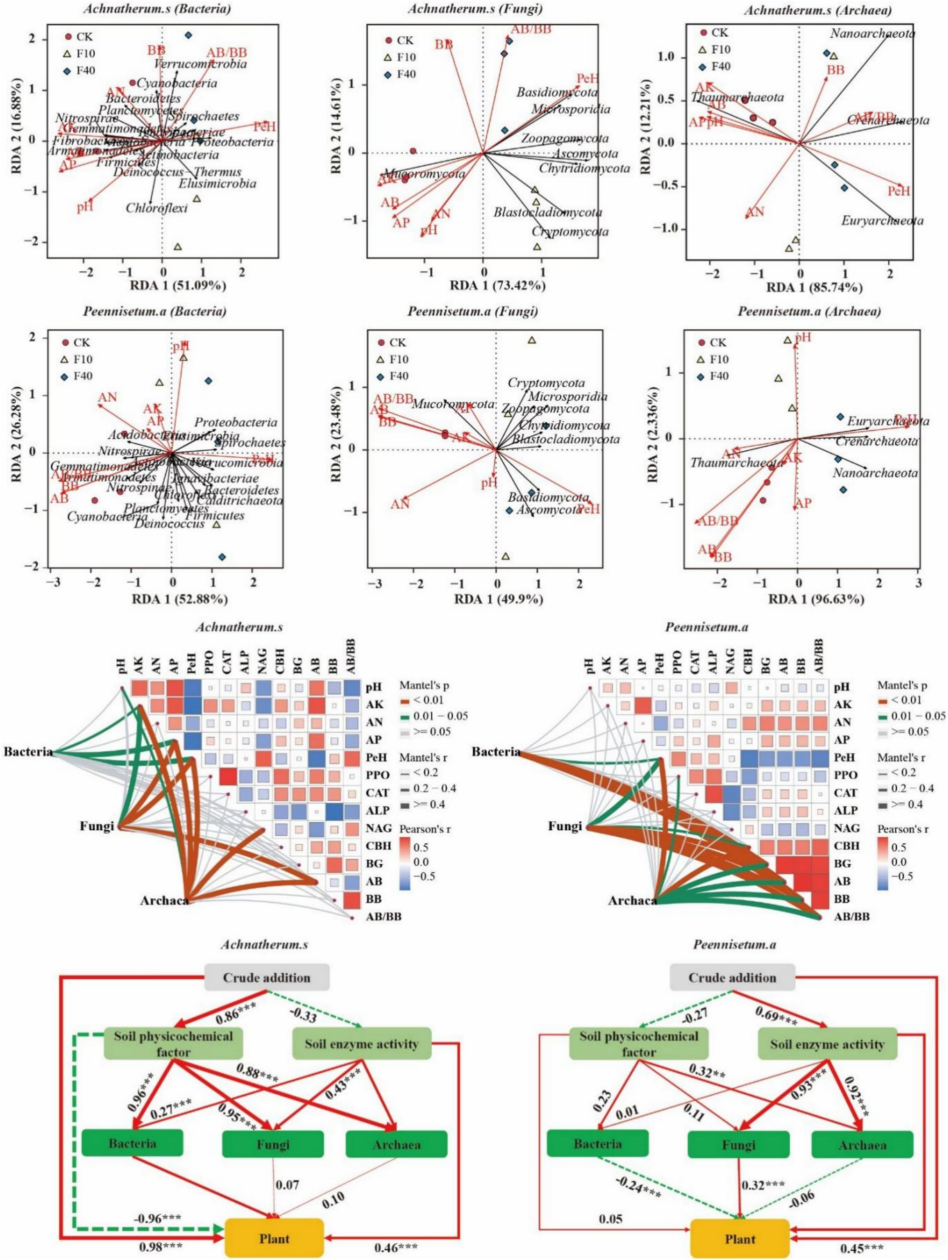
Correlation analysis of soil physicochemical factors and microorganisms. The structural equation model shows significant positive paths with red solid arrows, negative paths with green dashed arrows, and the numbers on the arrows represent standardized path coefficients. Additionally (*X*^2^ = 66.54, GFI = 0.91, RMSEA = 0.05 for *A. splendens*; *X*^2^ = 61.61, GFI = 0.89, RMSEA = 0.07 for *P. alopecuroides*). PPO, polyphenol oxidase activity; CAT, catalase activity; ALP, acid phosphatase activity; NAG, N-acetylglucosaminidase activity; CBH, cellulose hydrolysis enzyme activity; BG, β-glucosidase activity; AB, aboveground biomass; BB, belowground biomass; AK, available potassium in soil; AN, alkaline nitrogen in soil; AP, available phosphorus in soil; PEH, petroleum hydrocarbon.

Through correlation analysis (Figure 8), it was observed that in *A. splendens* soil, the bacterial taxa *Verrucomicrobia*, *Cyanobacteria*, and *Spirochaetes* exhibited positive correlations with BB, AA/BB, and PeH, respectively. Additionally, *Nitrospirae*, *Fibrobacteres*, *Gemmatimonadetes*, *Chloroflexi*, and *Fusobacteria* showed positive associations with AP, AK, and pH. In *P. alopecuroides* soil, the bacteria *Nitrospirae Acidobacteria* and *Proteobacteria* were positively correlated with AP AN AK and pH, while *Gemmatimonadetes Planctomycetes* and *Cyanobacteria* displayed positive relationships with AB BB and AA/BB. Furthermore, the fungal phyla *Basidiomycota*, *Zoopagomycota* and *Ascomycota* in both *A. splendens* and *P. alopecuroides* soils demonstrated positive correlations with PeH. *Mucoromycota* exhibited positive associations with AP AK and AB. The archaeal phylum *Thaumarchaeota* in both *A. splendens* and *P. alopecuroides* soils were positively correlated with AP, AK, and AB. Additionally, *Crenarchaea*, *Nanoarchaea* and *Euryarchaea* also displayed positive relationships with PeH.

Through Mantel analysis of the *A. splendens* community, it was determined that AK, AP, and PeH significantly influenced the bacterial community (*p* < 0.05), whereas AK, AP, PeH, and AB had a significant impact on the fungal community (*p* < 0.05). Additionally, pH, AK, AP, PeH, NAG and AB were found to significantly influence the archaeal community (*p* < 0.05). Regarding the *P. alopecuroides* community, Mantel test results indicated that PeH, BG, AB, BB, and AB/BB significantly influenced bacterial communities (*p* < 0.05). Furthermore, PeH, CBH, BG, AB, BB, and AB/BB had a significant impact on fungal communities (*p* < 0.05), and PeH, PPO, CBH, BG, AB, BB, and AB/BB significantly affected archaeal communities (*p* < 0.05).

Further analysis of *A. splendens* using structural equation modeling revealed a significant influence of crude oil addition on soil environmental factors (*p* < 0.05), which was significantly positively correlated with bacterial, fungal, and archaeal communities (*p* < 0.05). Additionally, crude oil addition, enzyme activity, and bacterial community were significantly positively associated with plant growth (*p* < 0.05), whereas environmental factors displayed a significant negative correlation with plant growth (*p* < 0.05). While environmental factors revealed a significant negative correlation with plant growth (*p* < 0.05). A Structural equation modeling of *P. alopecuroides* indicated that crude oil addition significantly influenced soil enzyme activity (*p* < 0.05), and soil environmental factors were significantly positively correlation with archaeal community (*p* < 0.05). Enzyme activity demonstrated a significant positive correlation with fungal and archaeal communities (*p* < 0.05). Furthermore, crude oil addition, enzyme activity, and fungal community were significantly positively correlated with plant growth (*p* < 0.05), while the bacterial community showed a significant negative correlation with plant growth (*p* < 0.05).

## 4 Discussion

### 4.1 Effects of crude oil addition on microbial community characteristics of *Achnatherum splendens* and *Pennisetum alopecuroides*

The variations in the relative abundance of bacterial phyla in *A. splendens* and *P. alopecuroides* under different levels of crude oil addition suggest differential responses to crude oil exposure. With increasing levels of crude oil addition, the overall relative abundance of dominant phyla in *A. splendens* shows an upward trend, while it shows a downward trend in *P. alopecuroides*. This disparity can be attributed to the higher tolerance of *A. splendens* to crude oil pollution, which allows it to maintenance or even enhance its growth at high concentrations, thereby increasing the relative abundance of bacterial phyla (Huang et al., 2022). Conversely, *P. alopecuroides* is more susceptible to crude oil pollution, resulting in growth inhibition and subsequent impacts on the bacterial community structure (Osman et al., 2020). *Proteobacteria* are known for their role in promoting plant growth, while Actinobacteria are involved in organic matter decomposition and nutrient cycling in soil ecosystems (Mitra et al., 2022). The addition of crude oil alters these dynamics, providing a competitive advantage to *Proteobacteria* while potentially inhibiting *Actinobacteria* due to the presence of crude oil derived compounds. *Ascomycota*, *Basidiomycota*, and *Mucoromycota* are common fungal phyla in soil, each fulfill specific ecological roles and functions. The reduction in relative abundance of the fungal phylum suggests that the addition of crude oil has modified the chemical and physical properties of the soil, which may adversely affect the growth and reproduction of fungi (Gałązka et al., 2020). *Thaumarchaeota* are typically involved in nitrification processes, whereas *Euryarchaeota* participate in denitrification and methane cycling (Li et al., 2024). As the amounts of crude oil added increase, a declining trend in the relative abundance of *Thaumarchaeota* is observed in both *A. splendens* and *P. alopecuroides* soils. In contrast, the relative abundance of *Euryarchaeota* shows a increasing trend. This phenomenon could be attributed to *Thaumarchaeota* generating energy through nitrification, while *Euryarchaeota* potentially use organic matter from from crude oil as an energy source during denitrification. In crude oil contaminated envirionments, inpaired energy production leads to a decline in *Thaumarchaeota* population, while their capacity to metabolize organic compounds in crude oil results in an increase in *Euryarchaeota* population (Liang et al., 2022).

Microorganisms do not exist in isolation but rather within complex networks of associations. Numerous studies have demonstrated that a more tightly interconnected network can facilitate the decomposition and utilization of nutrients (Lang et al., 2023). The introduction of crude oil significantly increases the number of edges and nodes in the bacterial, fungal, and archaeal networks in both *A. splendens* and *P. alopecuroides* soil, indicating that crude oil contamination enhances species interactions and networks complexity among these microorganisms. Under crude oil pollution conditions, microorganisms may enhance the survival capacity of the entire community by promoting inter-species cooperation. This adaptive change may involve mechanisms such as symbiosis and mutualism, thereby increasing network complexity (Borchert et al., 2021). In F10-treated *A. splendens* and *P. alopecuroides* soil bacterial communities, there is a strong cooperative relationship between species, however F40 treatment leads to a pronounced competitive relationship among species in soil bacterial communities. Low concentrations of crude oil may promote microbial synergistic degradation for mutual benefits; however, high concentrations can lead to resources scarcity, intense competition among microorganisms (Niu et al., 2020). In F40-treated *A. splendens* soil, both fungal and archaeal communities exhibit robust cooperative relationships. Conversely, CK-treated *A. splendens* and *P. alopecuroides* soil fungal and archaeal communities demonstrate competitive tendencies. These differences are likely due to variations in vegetation root secretions and growth habits under different conditions, which result in distinct types of cooperation or competition relationships (Bacon and White, 2016). Therefore, levering the synergistic interactions within microbial communities holds is crucial for soil bioremediation. By fostering the proliferation of cooperative microorganisms, the remediation rate of oil-contaminated soil can be accelerated, thereby enhancing the effectiveness of ecological restoration.

There is a significant positive correlation been found between the addition of crude oil, enzyme activity, and bacterial community with the growth of *A. splendens*. In contrast, a significant negative correlation exists between environmental factors and the growth of *A. splendens*. Similarly, a significant positive correlation is evident between crude oil addition, enzyme activity, and fungal community composition with the growth of *P. alopecuroides*; however, a notable negative correlation is also observed between bacterial community composition and the growth of *P. alopecuroides*. The introduction of crude oil may alter soil enzyme activity involved in its degradation, thereby mitigating its adverse effects on plant growth (Xun et al., 2015). Furthermore, active enzymes can enhance nutrient cycling in soil by promoting nutrient uptake through plant roots, thus boosting the growth of *A. splendens* (Chen et al., 2014). Changes in fungal communities have been shown to improved soil structure and nutrient cycling processes that ultimately benefit plant growth (Dai et al., 2021). Conversely, changes in bacterial communities may be linked to the production of plant hormones or metabolic processes related to crude oil degradation; certain bacterial communities associated with these processes can negatively impact plant growth leading to inhibition (Kuzina et al., 2021).

### 4.2 Effects of crude oil addition on soil ecosystem multifunctionality in *Achnatherum splendens* and *Pennisetum alopecuroides*

The soil ecosystem multifunctionality index serves as a critical metric for evaluating the health and stability of soil ecosystems, reflecting their capacity to provide ecosystem services such as nutrient cycling, carbon sequestration, and plant growth promotion (Zhang et al., 2020). The results revealed that the soil ecosystem multifunctionality index of *A. splendens* and *P. alopecuroides* showed a declining trend with increased crude oil contamination. Notably, the multifunctionality index in the F10 and F40 treatments was markedly lower compared with other treatments, indicating that elevated to the stress induced by on biotic process (Chakravarty et al., 2022). Toxic components found in crude oil, including polycyclic aromatic hydrocarbons and sulfides, can directly impair the soil microbial community in soils, resulting in reduced microbial activity and altered community structure, which in turn disrupts nutrient cycling, organic matter decomposition, and other associated ecological functions (Zhang et al., 2019). Path model analysis further elucidated the mechanisms by which bacterial, fungal, and archaeal communities influence soil ecosystem multifunctionality. Specifically, crude oil had a direct positive impact on the multifunctionality of the soil ecosystem in *A. splendens*, whereas it had a direct negative effect on the multifunctionality of the soil ecosystem in *P. alopecuroides*. These contrasting effects are likely due to the differing tolerance and recovery capabilities of the two plant species to crude oil pollution. Furthermore, crude oil contamination significantly modified the compositions of bacterial and fungal community compositions, which in turn influenced the composition of archaeal communities. These findings indicated that microbial community structure demonstrate a high degree of sensitivity to crude oil contamination and dynamically adjust in response to environmental changes. Within this framework, a direct positive correlation is observed between bacterial, fungal, and archaeal communities, suggesting potential interdependence within the soil ecosystem (Liang et al., 2019). Bacterial and fungal communities exhibit significant and direct negative influences on the multifunctionality of soil ecosystems in both *A. splendens* and *P. alopecuroides*. This phenomenon maybe attributed to various factors, such as reduced microbial activity due to crude oil exposure, changes in community composition, disruption in biogeochemical cycles, altered species competition and interactions, and secondary pollution. Collectively, these factors have contributed to a decline in the multifunctionality of soil ecosystems (Truskewycz et al., 2019). Conversely, archaeal communities play a vital role in enhancing the multifunctionality of soil ecosystems, likely due to their strong capacity for crude oil degradation and transformation in polluted environments (Singh and Choudhary, 2021). In summary, the introduction of crude oil significantly impacts the multifunctionality of soil ecosystems in both *A. splendens* and *P. alopecuroides* by altering in microbial community structure. This insight id crucial for understanding the long-term impacts of crude oil pollution on soil ecosystems and developing effective soil remediation strategies. It also underscores the importance of recognizing the contributions of microbial resources to maintain soil health and stability.

### 4.3 Ecological impacts and limitations of this study

In this study, we investigated the impact of crude oil addition on soil ecosystem stability and plant growth of *A. splendens* and *P. alopecuroides* by employing microbial co-occurrence networks and pathway analysis. Our results revealed a strong cooperative relationship between bacterial communities in *A. splendens* and *P. alopecuroides* soils under the F10 treatment, whereas a competitive relationship was observed under the F40 treatment. Additionally, under the F40 treatment, a significant cooperative association was found between fungal and archaeal communities in *A. splendens* soils; in contrast, under the F10 treatment, such cooperation was evident between fungal and archaeal communities in *P. alopecuroides* soils. Furthermore, increasing crude oil addition result in a progressive decline in the multifunctionality index of both *A. splendens* and *P. alopecuroides* soil ecosystems, with substantial reductions observed in both F10 and F40 treatments compared to other treatments. Bacterial and fungal communities exerted significant negative regulatory effects on the multifunctionality of soil ecosystem for *A. splendens* and *P. alopecuroides*; while archaeal communities demonstrated significant positive regulatory effects. Therefore, our study provides valuable support for conceptual models, energy flow dynamics, and nutrient transformations when evaluating the impact of crude oil contamination on different plants along with their associated soil environments’ multifunctionality (Schröder et al., 2018). Previous studies on the addition of crude oil to soil have primarily concentrated on physical, chemical, and biological indicators of soil, as well as the diversity and composition of microbial communities. However, the interaction between co-occurrence network patterns or structural characteristics of soil microbial communities under different crude oil additions and multifunctionality of soil ecosystems remains underexplored (Chen et al., 2023; Zhuang et al., 2023). Consequently, this study examined the stability and changes in ecosystem multifunctionality and ecological network structure of *A. splendens* and *P. alopecuroides* soils from an ecological perspective (Hu et al., 2023). The results revealed that under different crude oil addition scenarios, both connectivity and complexity of microbial networks were improved, indicating a more stable soil ecosystems with stronger resistance to environmental disturbances (Wang et al., 2019). Notably, that bacteria, fungi, and archaea played important roles in this process.

This study revealed the impact of varying crude oil concentrations on the bacterial, fungal, and archaeal community structure in soil inhabited by *A. splendens* and *P. alopecuroides*. It demonstrated that moderate crude oil addition may have adverse effects on microbial communities and overall ecosystem health. Therefore, excessive crude oil addition may exert detrimental effects on microbial communities and ecosystem health. Henceforth, future research should focus on comprehensive investigations into the integrated structure and function of soil micro-food webs, including fungi, bacteria, archaea, nematodes, and protozoa, to achieve a holistic understanding of the implications of crude oil additions on soil micro-food webs and ecosystem multifunctionality.

## 5 Conclusion

The study deepened our understanding of the mechanisms governing shifts in soil bacteria, fungi, and archaea communities in *A. splendens* and *P. alopecuroides* under varying crude oil addition conditions. Specifically, phyla *Proteobacteria*, *Actinobacteria*, and *Acidobacteria* were identified as dominant bacterial groups; *Ascomycota*, and *Basidiomycota* as dominant fungal groups, and *Thaumarchaeota* as the dominate archaeal groups. Ecological network analysis revealed robust cooperative relationships among bacterial species in *A. splendens* and *P. alopecuroides* soil under 10 g/kg crude oil addition. In contrast, strong cooperative relationships were observed among fungal and archaeal species in *A. splendens* soil under 40 g/kg crude oil addition; and in *P. alopecuroides* soil at10 g/kg crude oil addition. Additionally, both bacterial and fungal communities significantly negatively influenced the multifunctionality of *A. splendens* and *P. alopecuroides* soil ecosystems, while the archaeal community exhibited a positive regulatory effect. This study provides a theoretical basis for comprehending the multifunctionality of soil microbial systems under different levels and offers valuable insights for remediation of oil-contaminated soils.

## Data Availability

The names of the repository/repositories and accession number(s) can be found here: https://www.ncbi.nlm.nih.gov/, PRJNA1193340.

## Footnotes

^1^ http://cloud.majorbio.com

^2^https://github.com/OpenGene/fastp, version 0.20.0.

^3^https://github.com/voutcn/megahit, version 1.1.2.

^4^http://www.diamondsearch.org/index.php, version 0.8.35.

^5^http://www.diamondsearch.org/index.php, version 0.8.35.

^6^http://www.genome.jp/keeg/, version 94.2.
